# Secondary infection of *Fasciola gigantica* in buffaloes shows a similar pattern of serum cytokine secretion as in primary infection

**DOI:** 10.3389/fvets.2023.1109947

**Published:** 2023-04-20

**Authors:** Zhen Meng, Lele Zhai, Yanfeng Guo, Mengwei Zheng, Liang Li, Chongli Wen, Weiyu Zhang, Wenda Di

**Affiliations:** ^1^College of Animal Science and Technology, Guangxi University, Nanning, Guangxi, China; ^2^Guangxi Zhuang Autonomous Region Engineering Research Center of Veterinary Biologics, Guangxi University, Nanning, Guangxi, China; ^3^Guangxi Buffalo Research Institute, Chinese Academy Agricultural Sciences, Nanning, Guangxi, China

**Keywords:** cytokine, *Fasciola gigantica*, primary infection, secondary infection, susceptible

## Abstract

**Background:**

As a natural host of *Fasciola gigantica*, buffalo is widely infected by *F. gigantica*. Its impact on buffalo production has caused great losses to the husbandry sector, and repeat infection is non-negligible. In buffaloes experimentally infected with *F. gigantica*, primary and secondary infection have yielded the same rate of fluke recovery, indicating a high susceptibility of buffalo to *F. gigantica*, which contributes to the high infection rate. Determining the immunological mechanism of susceptibility will deepen the understanding of the interaction between *F. gigantica* and buffalo. Here, we explored the immune response of buffaloes against primary and secondary *F. gigantica* infection, with a focus on cytokines’ dynamics explored through serum cytokine detection.

**Methods:**

Buffaloes were assigned to three groups: group A (noninfected, *n* = 4), group B (primary infection, *n* = 3), and group C (secondary infection, *n* = 3). Group B was infected via oral gavage with 250 viable *F. gigantica* metacercariae, and group C was infected twice with 250 metacercariae at an interval of 4 weeks. The second infection of group C was performed simultaneously with that of group B. Whole blood samples were collected pre-infection (0 weeks) and at 1–6, 10, and 12  weeks after that. The serum levels of seven cytokines (IFN-γ, IL-4, IL-5, IL-10, IL-13, TGF-β, and IL-17) were simultaneously determined using ELISA and further analyzed.

**Results:**

In the present study, no significant changes in Th1-type cytokines production were detected in early infection, both in primary and secondary infections, while the Th2-type response was strongly induced. A comparison of primary and secondary infection showed no significant difference in the cytokine secretion, which may indicate that the re-infection at 4 weeks after primary infection could not induce a robust adaptive immune response. The full extent of interaction between buffalo and *F. gigantica* in re-infection requires further study.

## 1. Introduction

Fasciolosis, caused by *Fasciola hepatica* (in temperate zones) and *Fasciola gigantica* (in tropical zones), is one of the most widespread ruminant parasitic diseases and causes significant economic loss in the husbandry sector (1, 2). The tropical liver fluke, *F. gigantica,* affects the vitality and reproduction of infected buffaloes; given the high infection rate in buffaloes, this poses a serious threat to buffalo farming in Africa and Asia (3, 4).

Several studies have investigated Fasciola’s susceptibility and immunological mechanism in a primary infection (5–7). As *F. hepatica* infects cattle, its susceptible host, polarized Th2 is responsible for establishing the chronic phase and maintaining natural infection (6). Indonesian Thin Tail (ITT) sheep, as non-susceptible hosts, can resist infection by *F. gigantica*; an early Th1 immune response may be responsible for this (8). As susceptible hosts of *F. gigantica*, a mixed Th1/Th2 immune response was shown to have participated in the pathogenesis of *F. gigantica* infection in buffaloes (5, 7).

Explorations of the *Fasciola* secondary infection process have also been undertaken, indicating the consistency of host susceptibility to secondary and primary infection. Sheep, as a susceptible host of *F. hepatica*, were not resistant to the secondary infection of *F. hepatica* (9). However, ITT sheep were susceptible to the primary and secondary infection of *F. hepatica,* as flukes recovered from primary and secondary infections were similar to those of *F. hepatica*-susceptible sheep breeds (10). Nevertheless, susceptibility to secondary infection may vary depending on the time point of re-infection, as no resistance was detected in secondary infection of *F. hepatica* to its susceptible host, calves, 7 weeks after primary infection, while significant resistance was detected to secondary infection in calves 12 weeks after primary infection during the chronic phase; indicating the importance of the time-point in the establishment of secondary infection (11). ITT sheep acquire resistance to *F. gigantica* both in primary and secondary infection (12). Previous laboratory research found that buffaloes were susceptible to primary and secondary infection by *F. gigantica* 4 weeks after primary infection, as the flukes recovery rate was similar in primary and secondary infection (means of 21.2 and 23.5% burden, respectively) (13). Thus, it can be inferred that buffaloes are susceptible to secondary infection with *F. gigantica*, and that secondary infection at the fourth week cannot induce the robust adaptive immune response. However, the immunological precess behind this is unkown, and demonstration of this process will definitely guide the exploration of resistance process in other animal model, and helpful to vaccine development. Therefore, a secondary infection model was established, and the levels of the following seven serum cytokines were investigated in this study using enzyme-linked immunosorbent assay (ELISA): pro-inflammatory/Th1 [interferon (IFN)-γ], anti-inflammatory/Th2 (IL-4, IL-5, and IL-13), Treg [IL-10, transforming growth factor (TGF)-β], and Th17 (IL-17). Adaptive response induced by secondary infection could thus be explored, which may deepen the understanding of the susceptibility immunological mechanism in fluke infection and interaction between *F. gigantica* and buffalo.

## 2. Materials and methods

### 2.1. Maintenance of the metacercariae

Adult live *F. gigantica* collected from the gall bladder of buffalo (Guangxi, China) were washed with 37°C pre-warmed sterile RPMI 1640 media 3–4 times, and incubated in RPMI 1640 media supplemented with antibiotics (100 U/mL penicillin G, and streptomycin 0.1 mg/mL) and antimycotics (0.25 μg/mL amphotericin B) at 37°C for 2 h. Then the culture broth was centrifuged at 3,000 *g* for 30 min to collect the eggs. Eggs were incubated in dH_2_O for 3 weeks at room temperature protected from light, and miracidia were collected to infect *Galba pervia*. Each snail was infected with three miracidia through co-incubation in a sterile tissue culture plate for 1 h. Infected snails were then reared at 26°C for a further month, cercariae were adsorbed and encysted on 4 cm^2^ polythene strips, and then metacercariae was harvested and stored in dH_2_O at 4°C for later buffalo infection.

### 2.2. Experimental buffalo infection

**Figure 1:**
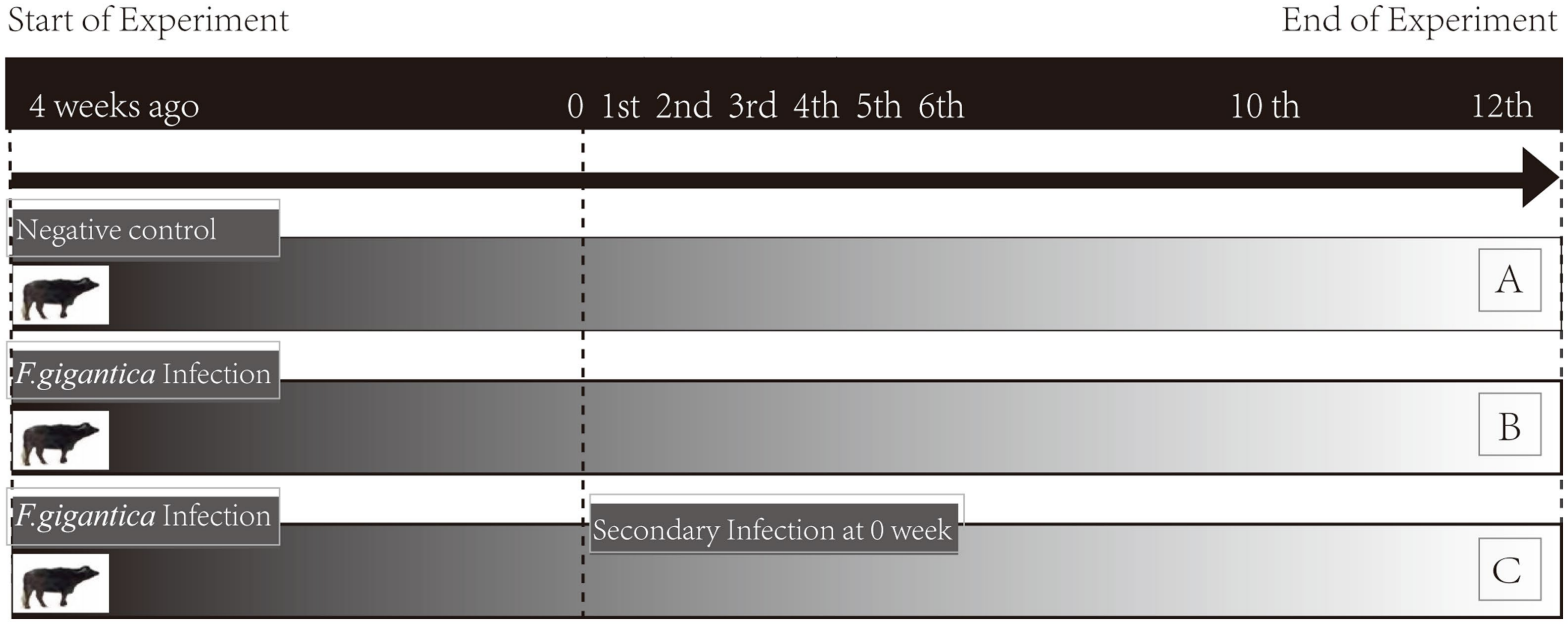
Experimental design. Noninfected buffaloes as negative control (group A); primary infected buffaloes (group B) received a single infection dose; secondary infected buffaloes (group C) received a primary infection and subsequent secondary infection at 4 wpi. Whole blood of groups A, B, and C were collected simultaneously pre-infection (week 0) and post-infection (weeks 1–6, 10, and 12).

This animal study was reviewed and approved by Ethics Committee of the College of Animal Science and Technology, Guangxi University. Ten buffaloes (6-month-old) of Murrah, Nili-Ravi, Mediterranean breeds, and their crossbred offspring with indigenous buffaloes in Guangxi, were randomly assigned to group A (noninfected, *n* = 4), group B (primary infection, *n* = 3), and group C (secondary infection, *n* = 3). They were stall-fed a balanced diet at the dairy of the Buffalo Research Institute, Chinese Academy of Agricultural Science, and verified to be free of parasitic infection through indirect ELISA based on Excretory-Secretory Products (*Fg*ESP) and coprological examination. Buffaloes in group C were given a gelatin capsule containing 250 *F. gigantica* metacercariae for primary infection and were reinfected with 250 metacercariae 4 weeks post-primary infection. Buffaloes in group B were infected by administration of 250 metacercariae orally at the same time of re-infection of group C. Blood samples were then collected to perform indirect ELISA in order to confirm the infection. To facilitate the description of group B and group C, the time-course of secondary infection for group C and primary infection for group B was conformably designated as 0 W, as seen in the following description (Figure 1).

### 2.3. Serum collection

Whole blood samples of group A, B, and C were collected simultaneously on a weekly basis from weeks 0–6, 10, and 12 post-infection (wpi). They were incubated at 37°C for 1 h for natural coagulation, and then the liquid was centrifuged at 3,000 rpm for 20 min at 4°C for supernatant collection. Subsequently, the serum layer was collected and stored at −80°C until use.

### 2.4. Determination of serum cytokines

The levels of cytokines, including IL-4, IL-5, IL-10, IL-13, IL-17, TGF-β, and IFN-γ, in serum were determined using ELISA (Bovine cytokine ELISA kit, Jiangsu Yutong, China) and conducted according to the manufacturer’s instructions. Briefly, the diluted standard substance and buffalo serum collected were added to the wells of the corresponding antibody pre-coated microtiter plate. It was then covered with a sealing membrane and incubated at 37°C for 30 min. Following this, the liquid was discarded, and each well washed five times (5 min per wash) with 200 μL washing solution. Afterward, 50 μL of enzyme-conjugate was added to each well, thoroughly mixed, covered with a sealing membrane, and incubated at 37°C for 30 min. Later, discard the liquid and wash five times with washing solution. For visualization, 50 μL of chromogenic agent A and 50 μL of chromogenic agent B were added to each well successively, shaken gently, and then incubated at 37°C in a dark place for 10 min. Afterward, 50 μL of stop solution was added to each well to terminate the reaction and the absorbance was then measured with a microplate reader (BIO-RAD, United States) at 450 nm (OD_450_).

### 2.5. Statistical analysis

The distribution of data was verified and subjected to following statistical analyses by GraphPad Prism 8. One-way ANOVA followed by Tukey’s test was used to evaluate differences within each group. Two-way analysis of ANOVA followed by Bonferroni’s multiple comparisons test was used to evaluate differences between the group B and C at the specific time courses during infection. *p* values were calculated, with *p* < 0.05 considered statistically significant.

## 3. Results

### 3.1. The establishment of infection

All buffaloes in group B and group C were challenged with *F. gigantica* seroconverted at **≥**2 wpi by ELISA based on *Fg*ESP, thereby indicating the establishment of infection. The autopsy at the end of the experiment (16 wpi) also verified the establishment, considering the fluke recovery rate was 21.2% for group B and 23.5% for group C (not shown). The numbers of flukes recovered were B1 (48/250), B2 (40/250), and B3 (71/250) in group B, and C1 (124/500), C2 (108/500), and C3 (120/500) in group C.

### 3.2. Secretion of Th1 type cytokine (IFN-γ)

**Figure 2:**
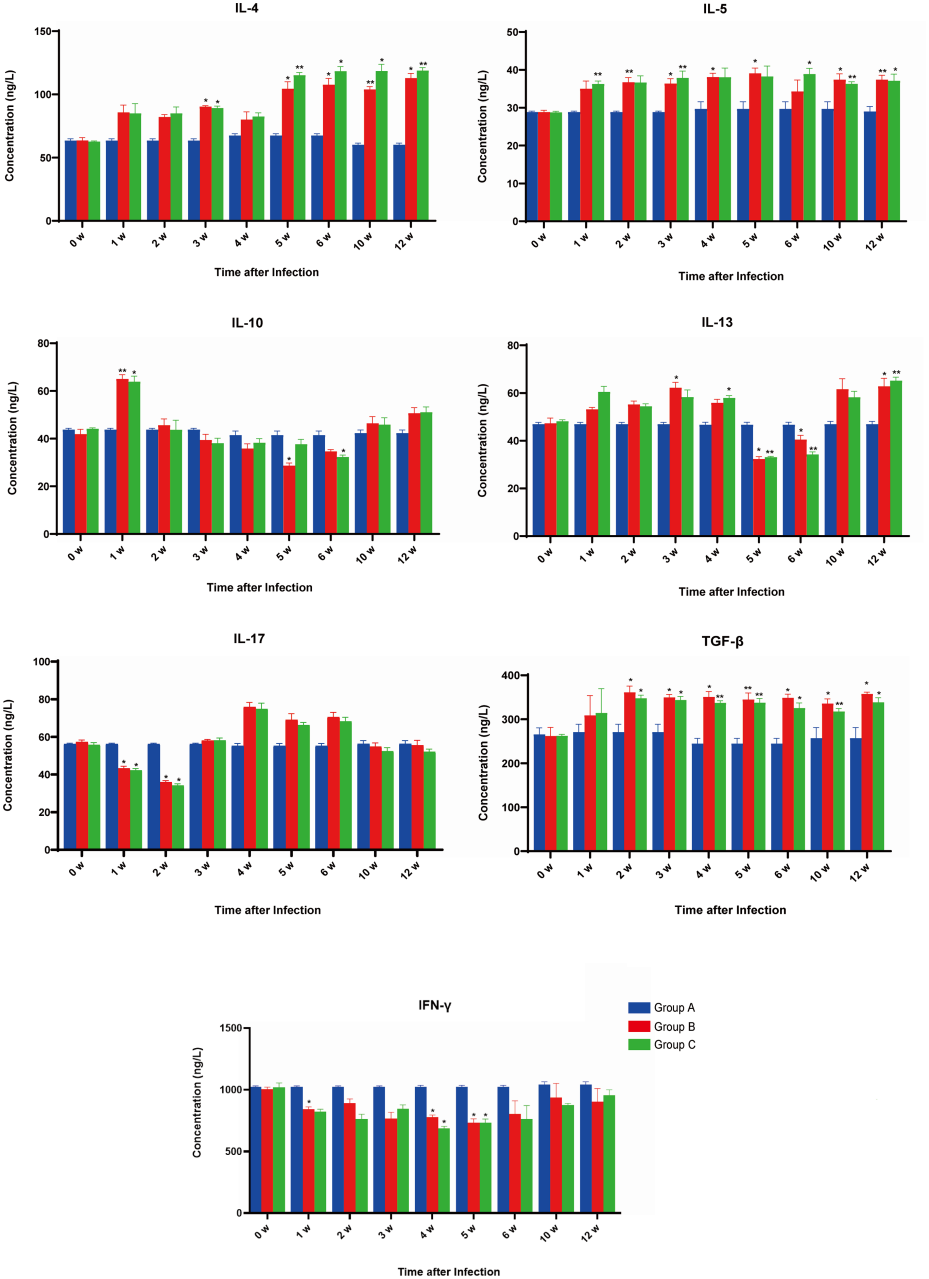
The effect of *Fasciola gigantica* on the levels of cytokine in the serum of primary and secondary experimentally infected buffaloes. The concentrations of seven cytokines were quantified pre-infection (week 0) and weekly after that for 8 weeks. Cytokine measurements were performed by ELISA kit and indicated in each panel. Bars represent the means ± SDs. Statistically significant (compared with pre-infection group) including *p* < 0.05 and *p* < 0.01 were indicated by asterisk (^*^) and (^**^) respectively.

The level of IFN-ϒ was stable in group A, and compared with week 0, no differences were detected. As for group B, all levels were decreased compared with pre-infection levels, with significant differences at 1, 4, and 5 wpi (*p* < 0.05). In group C, IFN-ϒ exhibited a similar trend as in group B, with 4 and 5 wpi levels significantly decreased (*p* < 0.05). Comparison of group B and group C indicated no differences throughout all weeks detected (Figure 2).

### 3.3. Secretion of Th2 type cytokines (IL-4, IL-5, and IL-13)

The level of IL-4 was stable in group A compared with week 0, as no differences were detected. However, the levels were increased for group B, compared with pre-infection, with significant differences at 3, 5, 6, 10, and 12 wpi. In group C, IL-4 exhibited a similar trend as in group B, with levels in 3, 5, 6, 10, and 12 wpi increasing significantly (*p* < 0.05). A comparison of group B and group C indicated no difference in the IL-4 secretion throughout all weeks detected (Figure 2).

The level of IL-5 was stable in group A, and compared with week 0, no differences were detected. Compared with group B, pre-infection levels increased throughout the whole period, with significant differences at 2, 3, 4, 5, 10, and 12 wpi (*p* < 0.05). In group C, IL-5 exhibited a similar trend as in group B, with significant differences at 1, 3, 6, 10, and 12 wpi (*p* < 0.05). A comparison of group B and group C indicated no differences throughout all weeks detected (Figure 2).

The level of IL-13 was stable in group A compared with week 0 and no differences were detected. For group B, compared with pre-infection, the level of IL-13 increased at 0–4 wpi, followed by a decline at 5–6 wpi, and then increased at 10 and 12 wpi, among which significant increases were detected at 3 and 12 wpi (*p* < 0.05), while significant decreases were detected at 5 and 6 wpi (*p* < 0.05). IL-13 in group C exhibited a similar trend to that of group B, with significant increases at 4 and 12 wpi (*p* < 0.05) and significant decreases in 5 & 6 wpi (*p* < 0.05). Comparison of group B and group C indicated no difference in IL-13 secretion throughout all weeks detected (Figure 2).

### 3.4. Secretion of Treg-type cytokines (IL-10 and TGF-β)

The level of IL-10 was stable in group A compared with week 0, with no differences detected throughout all weeks. For group B, compared with pre-infection, the level of IL-10 was increased at 1 wpi followed by attenuation; it was extremely increased (*p* < 0.01) at 1 wpi and significantly decreased (*p* < 0.05) at 5 wpi. IL-10 in group C exhibited a similar trend to that of group B, with a significant increase at 1 wpi (*p* < 0.05) and a significant decrease at 6 wpi (*p* < 0.05). When comparing groups B and C, no difference in IL-10 secretion was detected throughout all weeks (Figure 2).

The level of TGF-β was stable in group A compared with week 0 and no differences were detected throughout all weeks. For group B, compared with pre-infection, the level of TGF-β increased in all weeks detected and showed a significant difference at 2–6, 10, and 12 wpi (*p* < 0.05). In group C, TGF-β exhibited a similar trend as in group B, showing significant differences at 2–6, 10, and 12 wpi (*p* < 0.05). Comparing group B to group C showed no differences in TGF-β secretion throughout all weeks detected (Figure 2).

### 3.5. Secretion of Th17 type cytokine (IL-17)

The level of IL-17 was stable in group A compared with week 0, with no differences detected across all weeks. For group B, compared with pre-infection, the level of IL-17 fluctuated along with the prolonged infection, showing a significant decrease at 1 and 2 wpi (*p* < 0.05). In group C, IL-17 exhibited a similar trend to group B and showed significant decreases at 1 and 2 wpi (*p* < 0.05). Still, a comparison of group B to group C revealed no differences in TGF-β secretion throughout all weeks detected (Figure 2).

## 4. Discussion

In the present study, the dynamics of serum cytokines in primarily and secondarily *F. gigantica*-infected buffaloes were compared and investigated to explore the adaptive immunity of buffaloes and the susceptibility mechanism to *F. gigantica* present in buffaloes, which will deepen the understanding of the interaction between *F. gigantica* and buffalo. However, considering the limiting number of experimental animals here (*n* < 5), a larger scale of experimental animals should be explored to verify this precious process.

As a pro-inflammatory cytokine, IFN-γ functions as a host anti-parasitic infection, which can induce granuloma formation around damaged tissue to prevent parasite migration and development. Additionally, IFN-γ can also activate classically activated macrophages (M1), which can produce nitric oxide (NO) to kill the parasite during acute infection (14). In the present study, *F. gigantica* infection seemed to attenuate the level of IFN-γ, suggesting downregulation of Th1-type response in *F. gigantica* infection; this is in agreement with the observations of Zhang et al. (15), and may allow the parasite to evade host immune defense, thereby promoting its survival (6). No significant differences in IFN-γ cytokines levels were detected between groups B and C, demonstrating that secondary infection at 4 wpi did not induce significant changes in Th1-type cytokines production. Further work on Th1-type response will need to be explored.

The anti-inflammatory cytokine IL-4 can enhance Th2-type cell differentiation, promote fibrosis, and repair the injury site. During *F. gigantica* infection, elevated IL-4 in the early phase can activate the antibody-dependent cell-mediated cytotoxicity pathway (ADCC), producing harmful substances that eliminate fluke. In late-phase infection, the elevated IL-4 and IL-13 activate the alternative macrophage pathway (M2), which can produce molecules that are toxic to the fluke and contribute to fibrosis and tissue repair (14). Increased IL-4 in the early phase (1–6 wpi) may be associated with ADCC contributing to fluke elimination. Increased levels of IL-4 and IL-13 in late-phase infection (12 wpi) were detected in serum; these exhibited synergy, which may be associated with M2 activation for damaged tissue repair (16).

Finlay et al. (17) showed that *Fh*ESP could suppress Th1 and Th17 immune responses in the host by inducing the production of IL-5 and IL-33, thereby reducing the eliminating effects of the host against *F. hepatica*. The increased IL-5 shown in the present study may be associated with the immunomodulation of *Fg*ESP, thereby facilitating fluke survival. Donnelly reported that, during *F. gigantica* infection, IL-4 and IL-13 both work to inhibit Th1-type response and promote Th2-type response (18). Therefore, it can be speculated that elevation of Th2-type cytokines in this context may suppress Th1-type response, reducing buffaloes’ immune defense to a certain extent. No significant differences in Th2-type cytokines levels were detected between groups B and C, demonstrating that secondary infection at 4 wpi yields a similar Th2-type cytokine production pattern to that of primary infection.

IL-17 can promote the secretion of pro-inflammatory cytokine IL-1β and TNF-α, which subsequently can initiate and sustain an inflammatory response (19). Dowling reported that cathepsins, as well as glutathione of *Fh*ESP, could suppress inflammation-related responses by inhibiting IL-17 production, thus contributing to the survival of *F. hepatica* in the host (20). Decreased IL-17 at 1–2 wpi in this study may have been caused by fluke-derived components, which facilitated the establishment of infection by *F. gigantica* in the early phase. Increased IL-17 at 4–6 wpi was considered to inhibit infection with *F. gigantica*, as producing IL-17 can activate a Th1-type response and inhibit the Th2-type response (19). Furthermore, no significant differences were detected in IL-17 secretion between groups B and C throughout the study, demonstrating that secondary infection at 4 wpi failed to induce significant changes in Th17-type cytokine production.

During *F. hepatica* infection, IL-10 and TGF-β participated in IL-4 and IFN-γ production (21). IL-10 increased at 1 wpi, which presumably countered the migrating juvenile flukes, as IFN-ϒ was suppressed. Considering the dynamic changes of Th1-type and Th2-type cytokines, it can be argued that IL-10 and TGF-β may participate in maintaining the balance between the pro-inflammatory and anti-inflammatory responses in the interaction between buffalo and *F. gigantica* in different periods (22–24).

This dynamic study of *F. gigantica*-infected buffalo serum cytokines has revealed that, in the early stages of both primary and secondary infection, Th2/Treg dominated response was induced; this was manifested in increases of IL-4, IL-5, IL-10, IL-13, and TGF-β and reduction of IFN-γ and IL-17. A complex interaction between Th1/Th2/Treg/Th17 appeared to function in the following stages. In this assay, *F. gigantica* was found to downregulate Th1/Th17 response through Th2-type responses in early-stage infection, thereby allowing infection establishment in the host. Throughout the middle and late stages of infection, different cytokines functions were thus able to assist *F. gigantica* in surviving in buffaloes in the long term.

The prevention of Fasciolosis requires an understanding of the immune response during infections. Studies have suggested that a Th1 response shortly after fluke infection is associated with resistance to infection in resistant sheep, indicating that vaccine formulations should attempt to induce Th1 responses to enhance vaccine efficacy (25). Considering the vital role of Th1/Th2 in *F. gigantica* infection, understanding the Th1 and Th2 immune response can provide the basis for new vaccines and immune modulatory therapeutic development (26, 27). Here, the host reduced Th1, likely achieved by immune modulation of *F. gigantica*-derived molecules. As such, valuable and thorough analysis of the *Fg*ESP component that interacts with *F. gigantica*-infected buffalo serum, along with the infection process, especially during the early stage, will undoubtedly pave the way for vaccine candidate molecules and immune-modulatory therapeutic screening.

Here, seven cytokines were investigated, and no significant differences in secretion between primary and secondary infection were detected in all weeks tested. Furthermore, autopsies of buffaloes with primary and secondary infections indicated no significant difference in the fluke recovery rate between these groups (13). Combined with the dynamics of the cytokines, this verifies that the challenge infection could not induce resistance against *F. gigantica* in buffaloes. In line with this finding, it can be inferred that the lack of Th1-type response in secondary infection is correlated with the susceptibility of buffalo to secondary infection by *F. gigantica*.

## Data availability statement

The raw data supporting the conclusions of this article will be made available by the authors, without undue reservation.

## Ethics statement

The animal study was reviewed and approved by the Ethics Committee of the School of Animal Science and Technology, Guangxi University. Written informed consent was obtained from the owners for the participation of their animals in this study.

## Author contributions

WZ conceived and designed the experiments. ZM performed the experiments. LZ, ZM, and MZ wrote the manuscript. LL and CW performed buffalo maintenance. WD and YG reviewed the manuscript and contributed to the final submission. All authors contributed to the article and approved the submitted version.

## Funding

Project financial support was provided by the National Natural Science Foundation of China (Grant no. 31960706).

## Conflict of interest

The authors declare that the research was conducted in the absence of any commercial or financial relationships that could be construed as a potential conflict of interest.

## Publisher’s note

All claims expressed in this article are solely those of the authors and do not necessarily represent those of their affiliated organizations, or those of the publisher, the editors and the reviewers. Any product that may be evaluated in this article, or claim that may be made by its manufacturer, is not guaranteed or endorsed by the publisher.
